# Combination of endoscopic full-thickness resection and laparoscopic intragastric surgery for gastric submucosal tumor

**DOI:** 10.1055/a-2191-2310

**Published:** 2023-11-14

**Authors:** Atsuki Maeda, Kunihisa Uchita, Takehiro Iwasaki, Hiromichi Yamai

**Affiliations:** 1 Department of Gastroenterology, Kochi Red Cross Hospital, Kochi, Japan; 2 Department of Surgery, Kochi Red Cross Hospital, Kochi, Japan

Gastric submucosal tumors (SMTs) can be reliably resected in a minimally invasive manner using endoscopic full-thickness resection (EFTR) because, for example, they do not require mesenteric processing. However, there are many issues regarding suture methods, device costs, technique difficulty, and suturing certainty. Laparoscopic intragastric surgery allows secure suturing of the stomach wall from within the stomach by laparoscopy 1 . Although the combination of these two methods has not been reported, our experience indicates that it enables safer and less invasive treatment. We are convinced that this technique is particularly well suited for patients in whom oral tumor retrieval is impossible or suturing is difficult using only a flexible endoscope because a laparoscopic port is required.

CASE 1: A 76-year-old man had a 30-mm SMT with delle at the lesser curvature of the upper gastric body ( Fig. 1 ). Emergency surgery was required because of tumor hemorrhage. The patient strongly preferred minimally invasive EFTR instead of gastrectomy. The tumor was smoothly resected, but endoscopic ligation with O-ring closure (E-LOC method) 2 3 using a flexible endoscope alone was too difficult because of the high number of blood clots. Therefore, we switched to laparoscopic intragastric surgery. The gastric wall was tractioned to the umbilical wound, incised, and laparoscopically sutured in only 13 minutes ( Fig. 2 , Video 1 ).

**Fig. 1 a:**
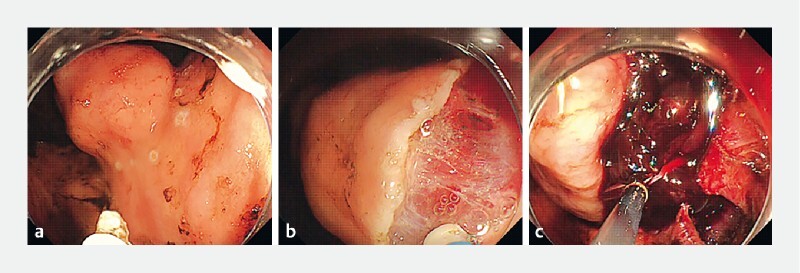
A 30-mm submucosal tumor with delle at the lesser curvature upper gastric body. **b** Tumor resection by a flexible endoscope. **c** Large number of blood clots obstructing vision during suturing.

**Fig. 2 a, b:**
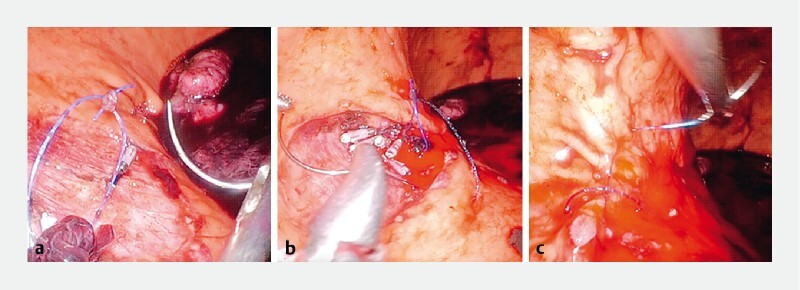
Surgical suturing under direct laparoscopic view with laparoscopic intragastric surgery. **c** Wound after surgical suture.

**Video 1** This video demonstrates the clinical utility of combining endoscopic full thickness resection and laparoscopic intragastric surgery to treat a gastric submucosal tumor.


CASE 2: A 75-year-old woman had a 25-mm SMT at the posterior wall of the middle gastric body. After the tumor had been resected by EFTR ( Fig. 3 ), the patient’s pharynx was lacerated during insertion of an overtube that was used to orally extract the tumor ( Fig. 4 ). We switched to laparoscopic intragastric surgery, which allowed for secure suturing and safe retrieval through the port ( Fig. 5 ). Both patients had good postoperative courses. From these experiences, we are convinced that the innovative combination of resection by EFTR and suture and retrieval by laparoscopic intragastric surgery is an effective method.

**Fig. 3 a:**
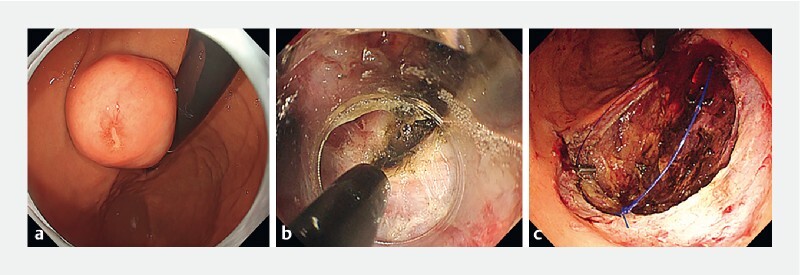
A 25-mm submucosal tumor at the posterior wall of the middle gastric body. **b** Tumor resection by a flexible endoscope. **c** Wound surface after resection by endoscopic full-thickness resection.

**Fig. 4 a:**
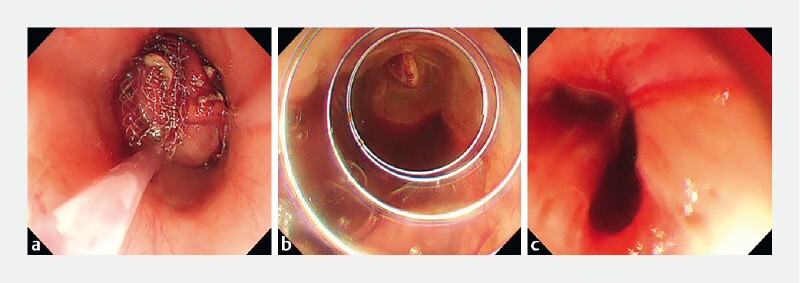
Attempt to retrieve lesions orally. **b** Placement of overtube. **c** Esophageal laceration associated with overtube placement.

**Fig. 5 a, b:**
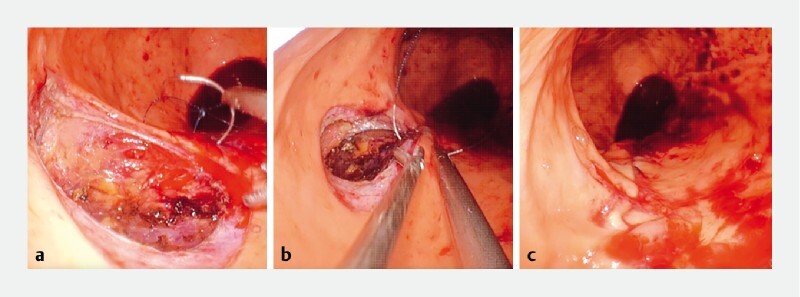
Surgical suturing under direct laparoscopic view with laparoscopic intragastric surgery. **c** Wound after surgical suture.

Endoscopy_UCTN_Code_CCL_1AB_2AD_3AB

## Notes

Endoscopy E-Videos

https://eref.thieme.de/e-videos
*E-Videos* is an open access online section of the journal *Endoscopy* , reporting on interesting cases and new techniques in gastroenterological endoscopy. All papers include a high-quality video and are published with a Creative Commons CC-BY license. Endoscopy E-Videos qualify for HINARI discounts and waivers and eligibility is automatically checked during the submission process. We grant 100% waivers to articles whose corresponding authors are based in Group A countries and 50% waivers to those who are based in Group B countries as classified by Research4Life (see: https://www.research4life.org/access/eligibility/ ). This section has its own submission website at https://mc.manuscriptcentral.com/e-videos
